# Ectopic Pancreas: A Chameleon of Abdominal Symptoms

**DOI:** 10.7759/cureus.106328

**Published:** 2026-04-02

**Authors:** Rakshana Munusamy, Evangelia Florou, Rajendran Vellaisamy, Evangelos Prassas, Parthi Srinivasan, Raj Srirajaskanthan, Andreas Prachalias

**Affiliations:** 1 Hepato-Pancreato-Biliary Surgery, King's College Hospital, London, GBR; 2 Gastroenterology, King's College Hospital, London, GBR; 3 Hepato-Pancreato-Biliary Surgery and Liver Transplantation, London Bridge Hospital, London, GBR

**Keywords:** bowel obstruction, duodenum, ectopic pancreas, gastrointestinal stromal tumour, heterotopic pancreas

## Abstract

Ectopic pancreas, also known as heterotopic pancreas, is a congenital anomaly characterised by pancreatic tissue located outside its normal anatomical location, without structural, vascular, or ductal continuity with the native pancreas. Although often asymptomatic, an ectopic pancreas may present as an abdominal mass and radiologically mimic other pathologies, resulting in diagnostic uncertainty and frequently leading to surgical intervention.

We report two cases: a 49-year-old man who presented with abdominal discomfort and fullness and was found to have a 2 cm lesion at the duodenojejunal flexure. Workup failed to delineate the nature of the lesion, and the patient underwent resection, revealing ectopic pancreas. A 33-year-old female presented with symptoms of small bowel obstruction. Extensive workup, including capsule endoscopy, revealed an intraluminal polypoid lesion, and underwent laparotomy and resection. In both cases, histopathology confirmed ectopic pancreas.

These cases highlight the diagnostic challenges that ectopic pancreas may pose and emphasise its role as a true “chameleon” of abdominal pathology. Its variable clinical and radiological presentations underscore the importance of considering ectopic pancreas in the differential diagnosis of symptomatic gastrointestinal masses.

## Introduction

Ectopic pancreas, also referred to as heterotopic pancreas, is defined as pancreatic tissue located outside its normal anatomical location without direct structural, vascular, or ductal continuity with the native pancreas [1,2]. This developmental anomaly is believed to arise during embryogenesis due to abnormal migration or separation of pancreatic tissue from the primitive foregut. During rotation and fusion of the dorsal and ventral pancreatic buds, small fragments of pancreatic tissue may become displaced along the gastrointestinal tract, where they retain pancreatic differentiation but lack anatomical, vascular, and ductal continuity with the orthotopic pancreas [1,2]. This embryologic misplacement explains the predilection of ectopic pancreas for the stomach, duodenum, and proximal jejunum, which are closely related to the foregut and midgut during development [2,3].

Histologically, ectopic pancreas is commonly classified according to the Heinrich classification system [1,4]. Type I consists of pancreatic acini, ducts, and islets of Langerhans (complete pancreatic tissue). Type II contains pancreatic acini and ducts without islet cells. Type III (also known as the adenomyoma type) is composed predominantly of ducts with few or absent acini and no islets.

The reported incidence of ectopic pancreas varies widely, ranging from 0.5% to 13% in autopsy and endoscopic series [1,3]. The most common sites include the stomach, duodenum, and proximal jejunum, accounting for approximately 70-90% of cases [2,3]. Less frequently, an ectopic pancreas may be found in the ileum, Meckel’s diverticulum, gallbladder, spleen, or mesentery.

Most cases are asymptomatic and detected incidentally during imaging, endoscopy, or surgery performed for unrelated indications. However, ectopic pancreas may occasionally present with abdominal pain, gastrointestinal bleeding, obstruction, pancreatitis, and, rarely, malignant transformation has been reported [4,5].

In this report, we present two cases of symptomatic ectopic pancreas with distinct clinical manifestations; one mimicking a subepithelial neoplastic lesion with diagnostic uncertainty despite multimodal imaging, and another presenting as a lead point for small bowel intussusception. These cases highlight the diagnostic challenges and variable clinical presentations of ectopic pancreas, justifying its characterisation as a clinical “chameleon.”

## Case presentation

Case 1

A 49-year-old man presented with abdominal discomfort and a sensation of fullness. His past medical history was significant for supraventricular tachycardia managed medically. He had no history of prior abdominal surgery and no significant gastrointestinal history. His performance status was 0.

**Figure 1:**
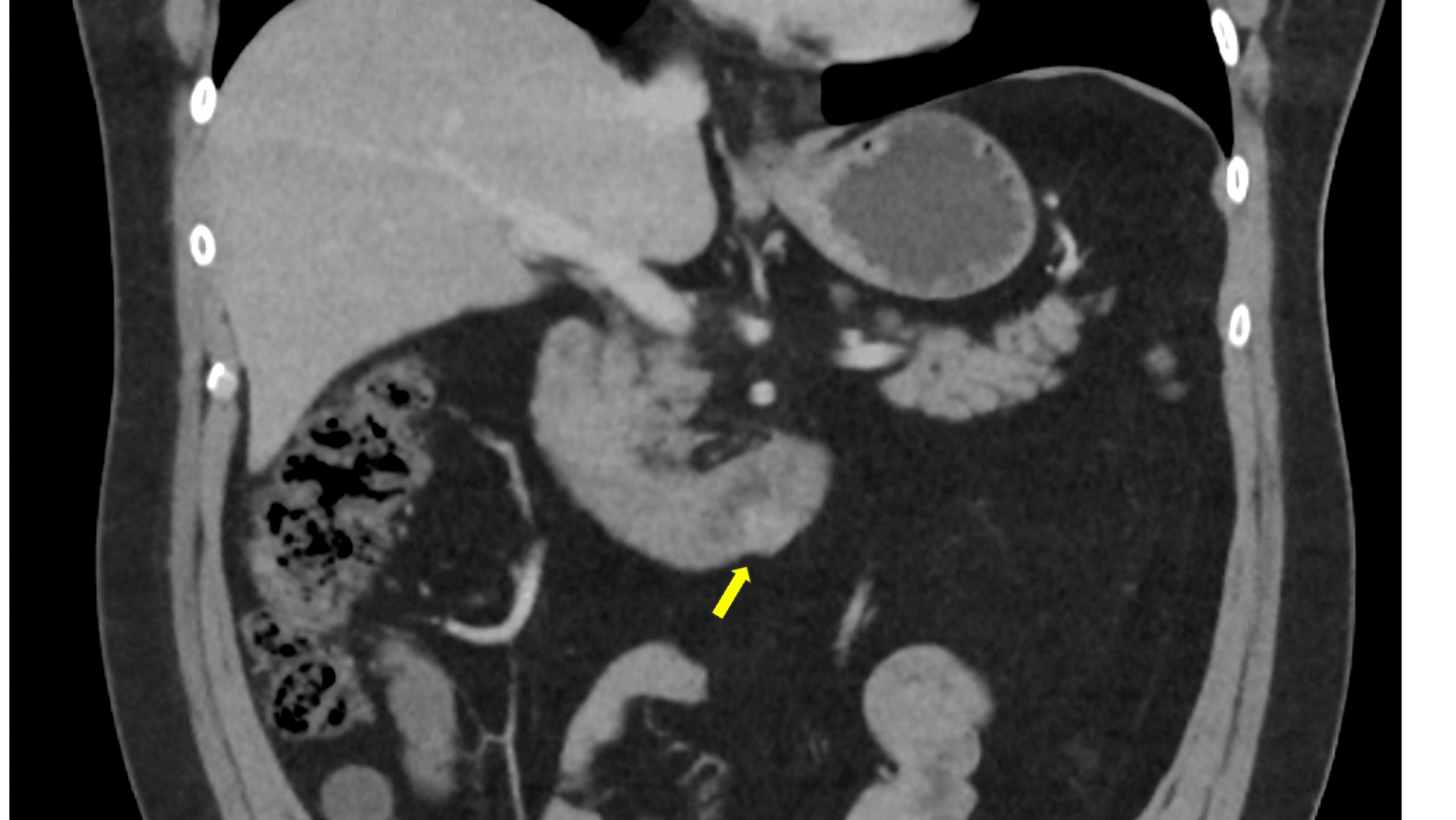
Case 1: Coronal CT view of ectopic pancreas. Contrast-enhanced CT of the abdomen, coronal view, demonstrating the well-defined subepithelial soft tissue lesion (yellow arrow) arising from the posterior wall at the junction of the third and fourth parts of the duodenum. The lesion shows homogeneous enhancement and projects into the lumen without associated obstruction or surrounding inflammatory changes. CT: computed tomography

**Figure 2:**
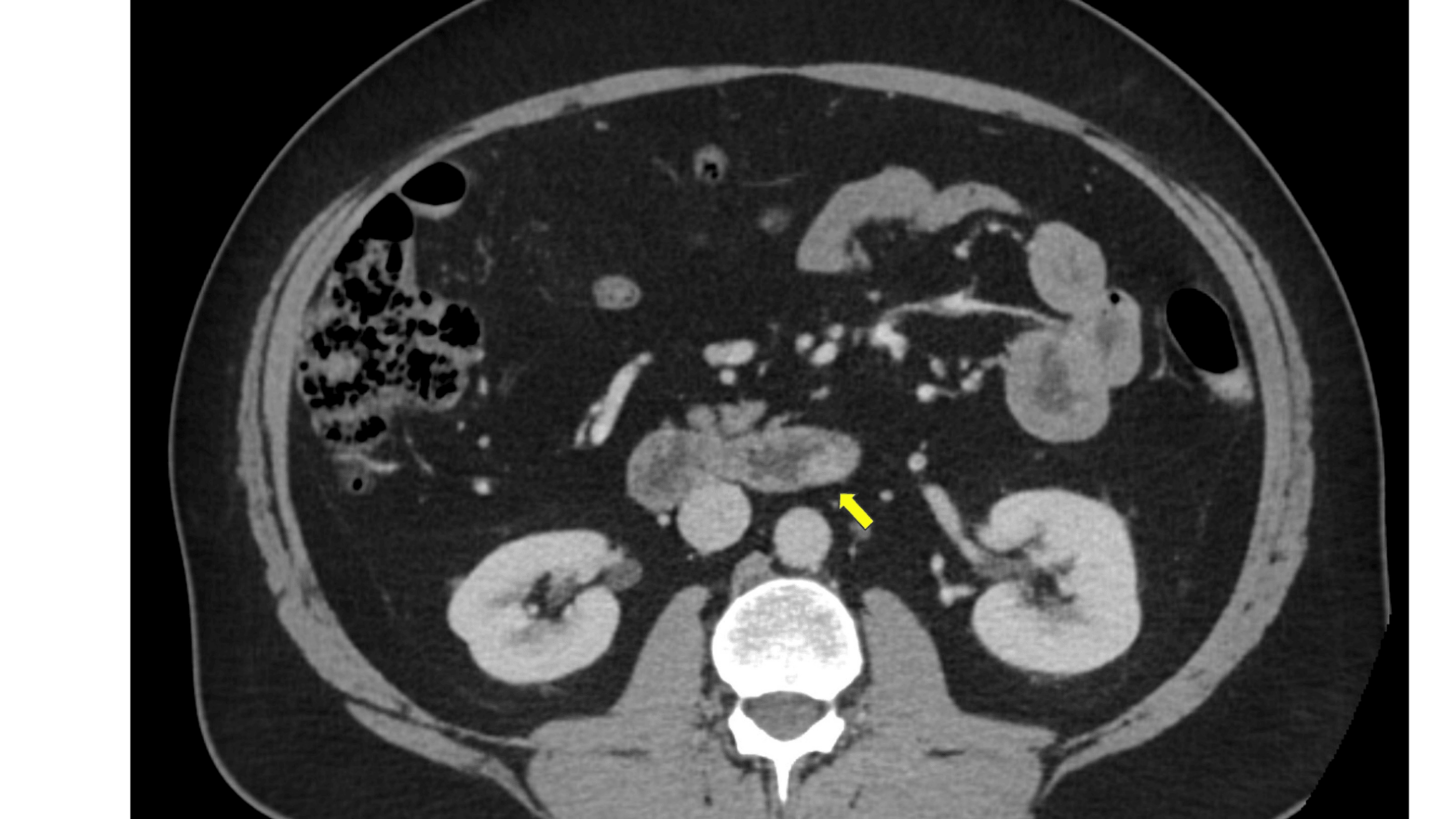
Case 1: Axial CT view of ectopic pancreas. Contrast-enhanced CT of the abdomen, axial reconstruction, demonstrating a well-circumscribed, approximately 2 cm eccentric soft tissue lesion (yellow arrow) arising from the posterior wall at the junction of the third and fourth parts of the duodenum (D3/D4). The lesion projects into the lumen without evidence of obstruction or surrounding inflammatory change and was radiologically suspicious for a GIST. CT: computed tomography; GIST: gastrointestinal stromal tumour

Contrast-enhanced computed tomography (CT) of the abdomen demonstrated a well-defined, approximately 2 cm eccentric soft tissue lesion arising from the posterior wall at the junction of the third and fourth parts of the duodenum. The lesion, measuring approximately 20 mm, projected into the lumen without evidence of obstruction or surrounding inflammatory changes (Figures 1-2). Based on imaging characteristics and anatomical location, the lesion was considered suspicious for a gastrointestinal stromal tumour (GIST).

**Figure 3:**
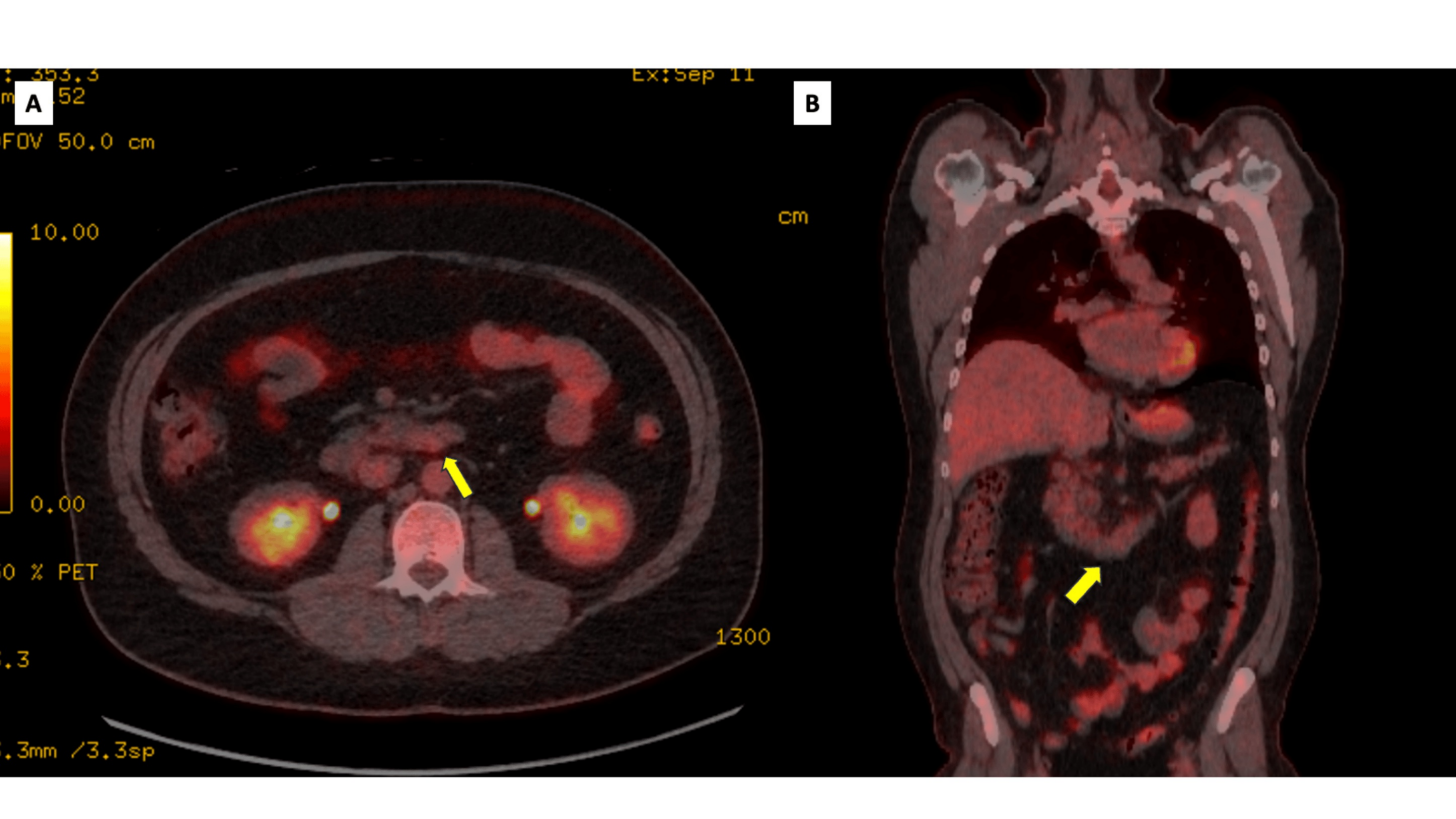
Case 1: FDG-PET imaging of ectopic pancreas. FDG-PET images demonstrating no significant metabolic uptake within the duodenal lesion at the D3/D4 junction (yellow arrow), indicating absence of hypermetabolic activity. (A) Axial view. (B) Coronal view. FDG-PET: fluorodeoxyglucose-positron emission tomography

Enteroscopy was performed, which did not demonstrate any intraluminal abnormality, suggesting that the lesion was subepithelial in origin, highlighting the limitations of endoscopic evaluation in such cases. Further evaluation with positron emission tomography-computed tomography (PET-CT) demonstrated no fluorodeoxyglucose (FDG) uptake within the lesion (Figure 3). Serum tumour markers, including carcinoembryonic antigen (CEA), carbohydrate antigen 19-9 (CA 19-9), and alpha-fetoprotein (AFP), were all within normal limits (Table 1).

**Table 1:** Case 1: Serum tumour markers. Serum tumour markers were assessed and found to be within normal limits. CEA: carcinoembryonic antigen; CA 19-9: carbohydrate antigen 19-9; AFP: alpha-fetoprotein

Marker	Result	Reference range
CEA	3.1	<5 ng/mL
CA 19-9	26	<37 U/mL
AFP	2	<10 ng/mL

In view of imaging findings and negative PET-CT, the lesion remained indeterminate. The case was discussed at a hepatopancreatobiliary (HPB) multidisciplinary meeting (MDM), and given persistent concern for GIST, surgical resection was recommended to establish a definitive diagnosis and exclude malignancy.

On laparotomy, a firm, approximately 2 cm lesion was identified at the junction of the third and fourth parts of the duodenum, involving more than 50% of the bowel circumference. Segmental resection of the affected portion of the duodenum and proximal jejunum was performed, followed by side-to-side duodeno-jejunal anastomosis. There were no intraoperative complications.

The postoperative course was uneventful. A postoperative contrast gastrointestinal study performed during the same admission demonstrated no evidence of anastomotic leak. Oral intake was gradually resumed, and the patient was discharged.

**Figure 4:**
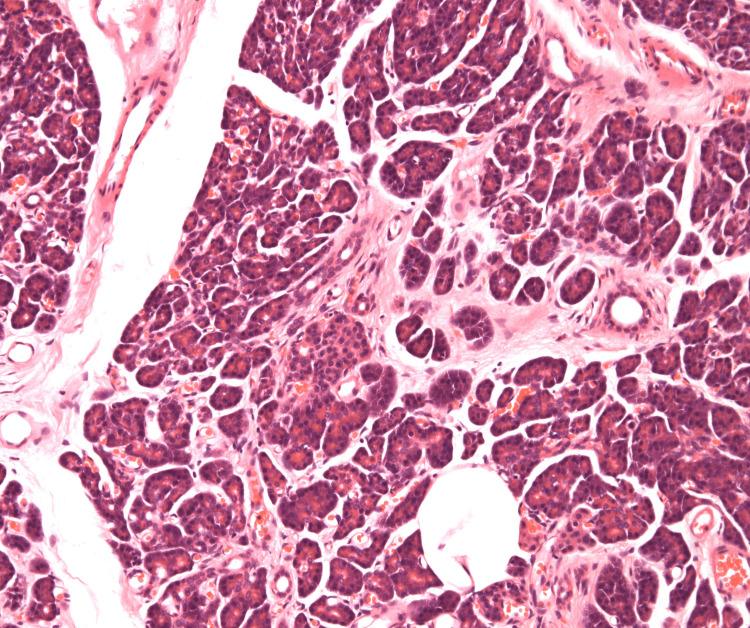
Case 1: Haematoxylin and eosin-stained section of ectopic pancreas. Haematoxylin and eosin-stained section demonstrating mature pancreatic tissue within the submucosal layer, composed of well-formed acini, ducts, and islets of Langerhans, consistent with Heinrich type I heterotopic pancreas. Original magnification ×100.

Histopathological examination demonstrated a well-circumscribed nodular lesion measuring 24 mm composed of mature pancreatic tissue, including pancreatic acini, ducts, and islets of Langerhans, consistent with Heinrich type I ectopic pancreas [1,4]. The ectopic pancreatic tissue involved the submucosal, muscular, and subserosal layers. There was no evidence of dysplasia or malignancy (Figure 4). Surgical margins were clear. At outpatient follow-up, the patient demonstrated excellent postoperative recovery. He reported restoration of normal appetite and satisfactory bowel function.

Case 2

**Figure 5:**
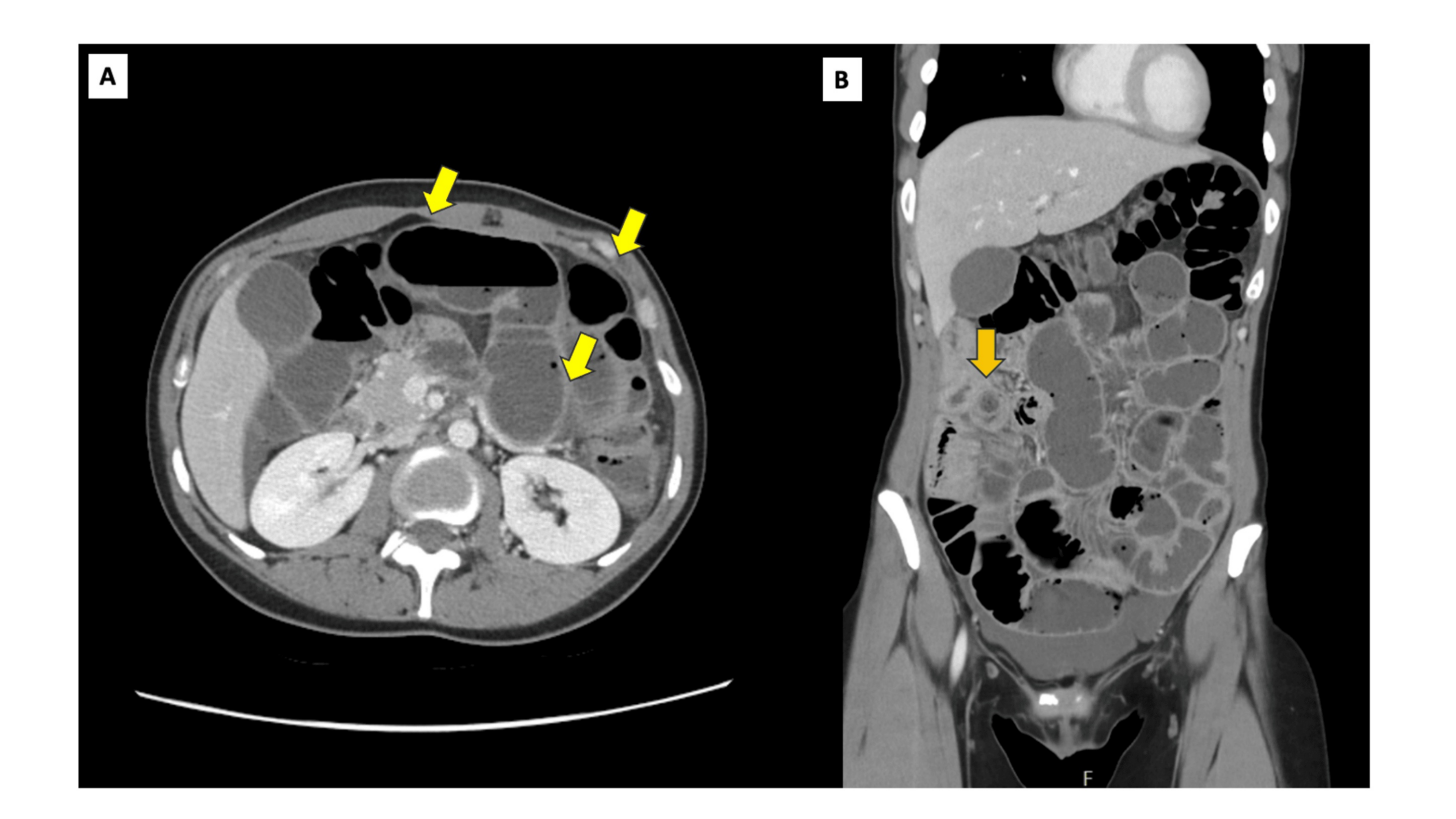
Case 2: CT images of small bowel obstruction. (A) Axial CT image demonstrating multiple dilated small bowel loops (yellow arrows), consistent with small bowel obstruction. (B) Coronal CT reconstruction confirming small bowel obstruction. On retrospective review, a subtle concentric configuration (orange arrow) may correspond to the site of intussusception. CT: computed tomography

A 33-year-old female initially presented with symptoms of bowel obstruction and was acutely admitted to a local hospital. CT imaging demonstrated evidence of small bowel obstruction (Figure 5). She was managed conservatively on this occasion. In view of her young age and absence of previous surgical history, the case was referred for discussion at the HPB MDM.

**Figure 6:**
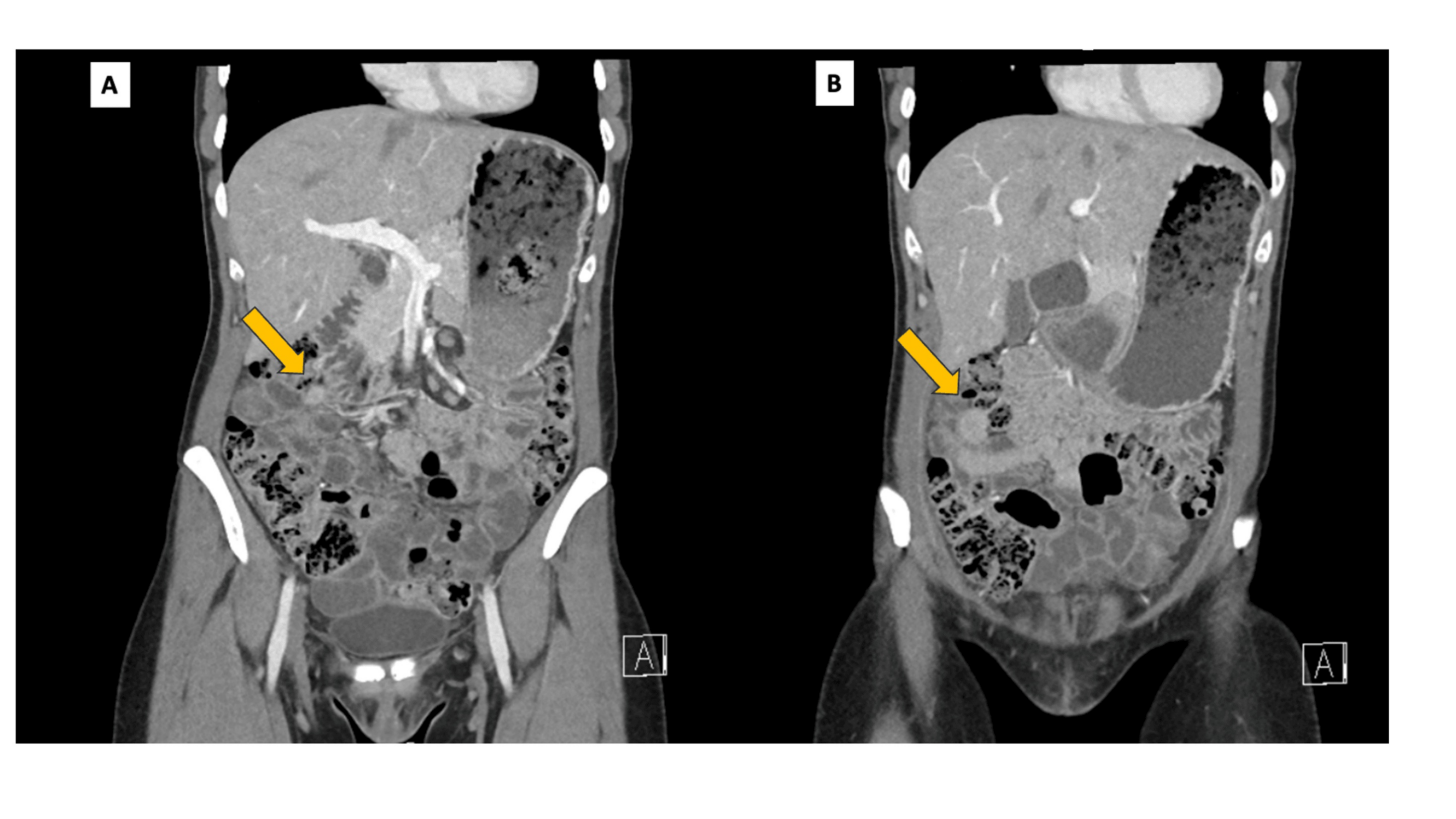
Case 2: CT images of ectopic pancreas. (A) Coronal CT image obtained after recovery from the initial episode of small bowel obstruction and prior to definitive surgical intervention (preoperative CT), demonstrating small bowel loops without a clearly definable focal mass at the time of prospective interpretation. (B) Coronal CT reconstruction again failing to clearly characterise a discrete lesion. On retrospective review, a subtle focal concentric configuration (orange arrow) may correspond to the site of intussusception identified intraoperatively. CT: computed tomography

An MRI scan identified a lesion within the small bowel, although this was not well characterised. A subsequent CT scan did not clearly demonstrate a definable lesion (Figure 6). Following the transient episode of small bowel obstruction that resolved clinically, and in the absence of persistent radiological evidence of obstruction, capsule endoscopy was considered appropriate to evaluate for an underlying small bowel lesion. The latter confirmed the presence of a large polypoid lesion in the mid-to-distal small bowel.

**Figure 7:**
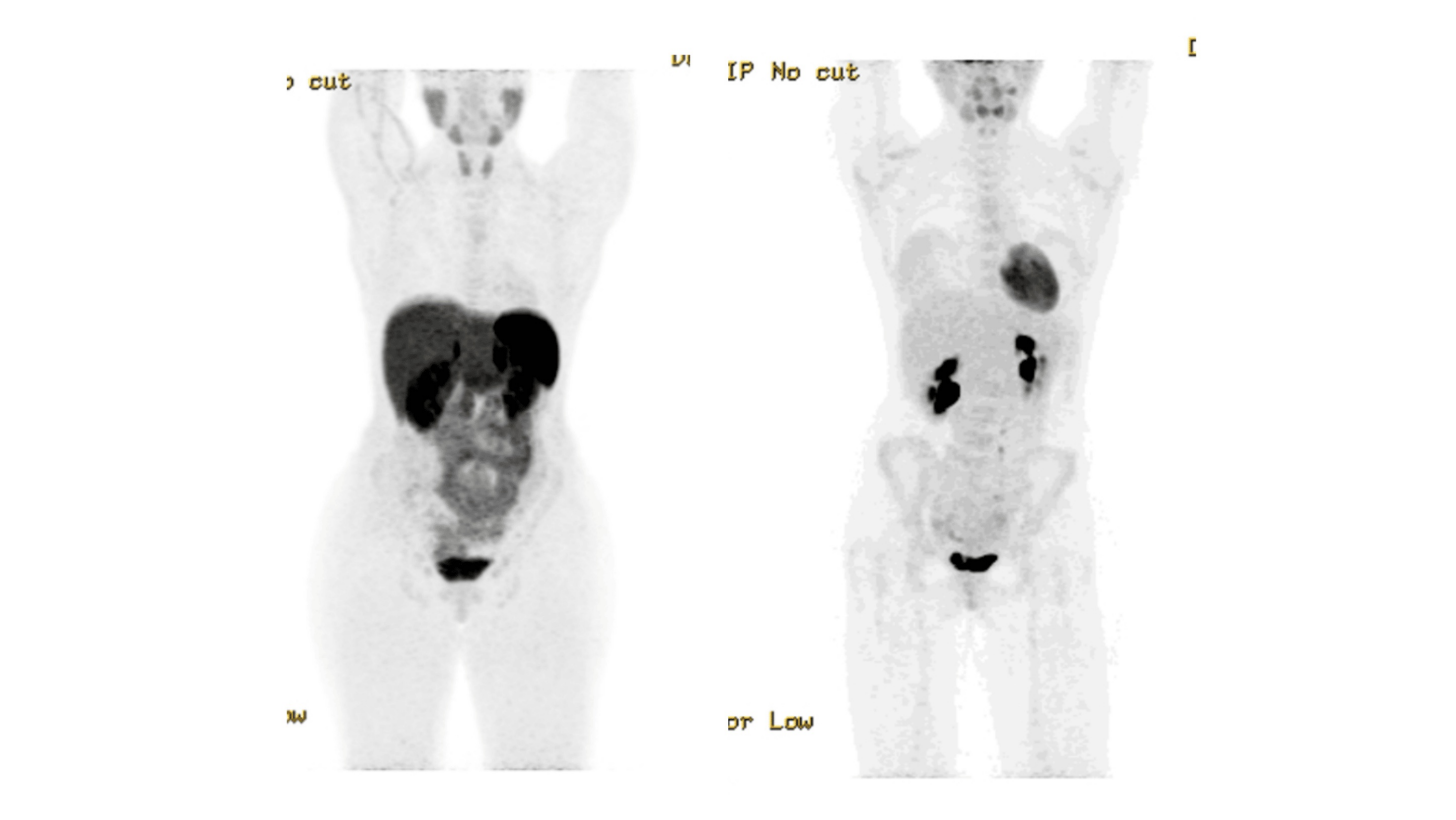
Case 2: FDG-PET image of the small bowel lesion. FDG-PET image demonstrating no abnormal metabolic avidity within the small bowel lesion. FDG-PET: fluorodeoxyglucose-positron emission tomography

**Figure 8:**
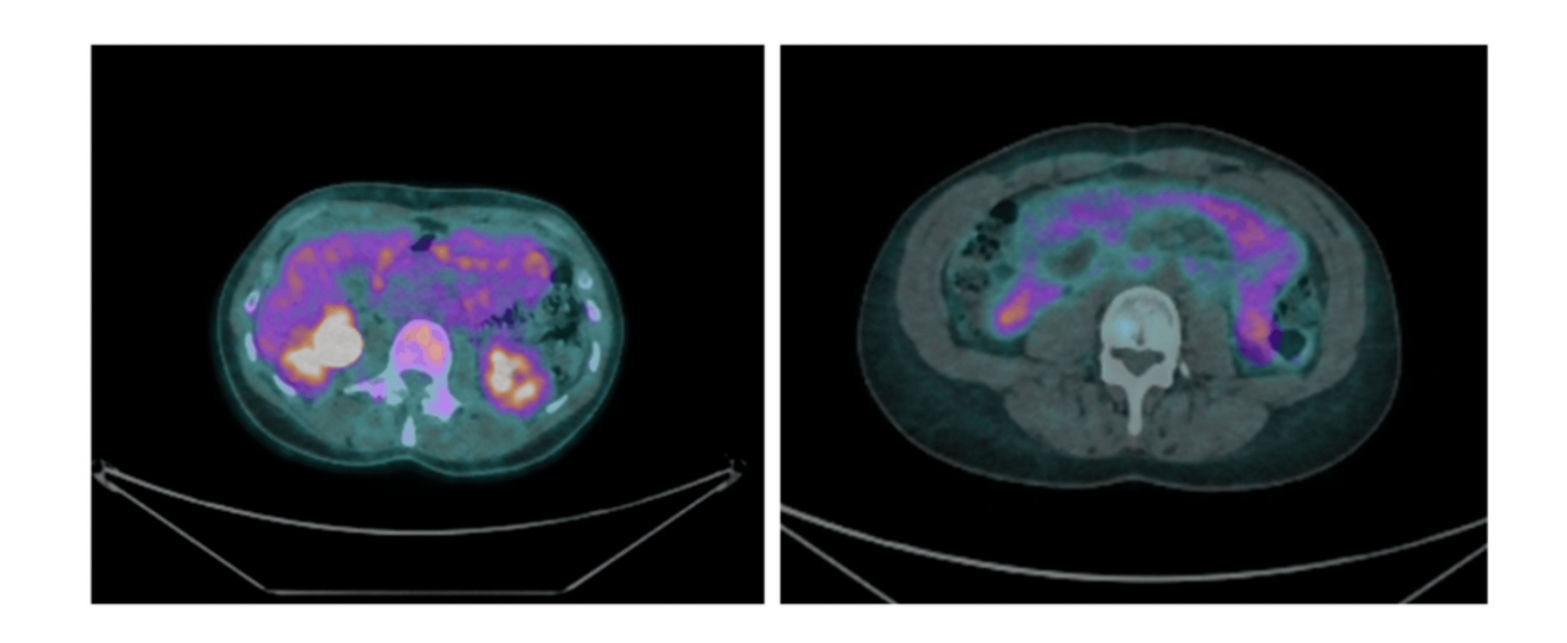
Case 2: DOTATATE PET images. DOTATATE PET image demonstrating no abnormal somatostatin receptor avidity within the small bowel lesion, arguing against a NET. DOTATATE PET: positron emission tomography with gallium-68 DOTATATE; NET: neuroendocrine tumour

Given her persistent symptoms, surgical resection was recommended. A FDG-PET scan demonstrated no metabolic avidity within the lesion. Workup was completed with a gallium-68 DOTATATE positron emission tomography (DOTATATE PET) scan to exclude neuroendocrine tumour (NET), which was also negative (Figures 7-8).

At laparotomy, an intussusception was identified in the mid-ileum. Following reduction of the intussusception, an intraluminal lesion was palpated. The procedure was completed with segmental small bowel resection and a side-to-side ileo-ileal anastomosis. She made an uneventful recovery.

**Figure 9:**
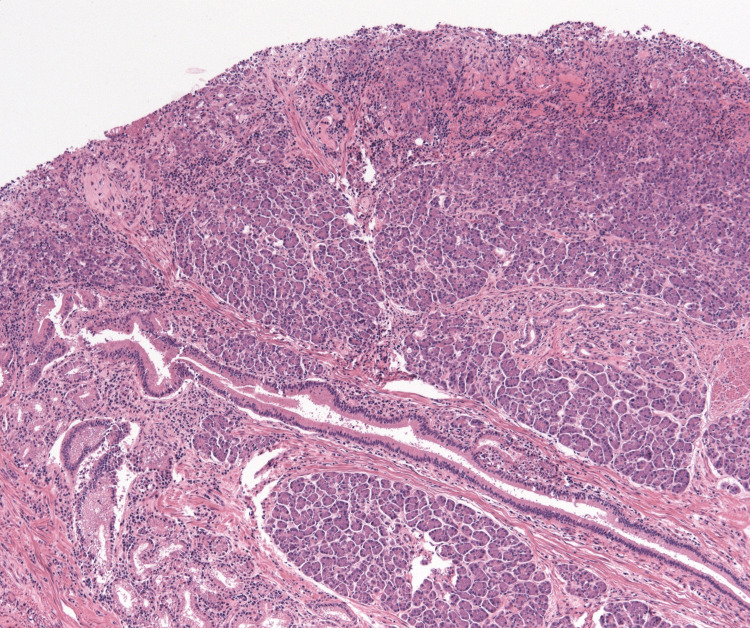
Case 2: Haematoxylin and eosin-stained section of ectopic pancreas. Haematoxylin and eosin-stained section demonstrating erosive surface epithelium with underlying submucosal pancreatic tissue composed of acini and ducts, consistent with heterotopic pancreas. Original magnification ×40.

Histological examination demonstrated that the intussuscepted segment consisted of an inverted intestinal wall with partially erosive surface epithelium. At the tip of the polypoid lesion, heterotopic pancreatic tissue was identified. Pyloric gland-like elements were also present (Figure 9). There was no evidence of dysplasia or malignancy. The background mucosa was unremarkable, and the mesenteric lymph nodes showed no pathological abnormality.

Both cases had a brief and uneventful postoperative course. No recurrence or related long-term symptoms were identified within the available short follow-up period. These two cases illustrate the diverse clinical presentations of ectopic pancreas, ranging from an indeterminate subepithelial duodenal lesion suspicious for GIST to a lead point for intussusception causing small bowel obstruction.

## Discussion

The majority of ectopic pancreas lesions are asymptomatic and are discovered incidentally during endoscopy, imaging, or surgery performed for unrelated indications [1,4]. When symptomatic, presentation depends largely on lesion size, anatomical location, and associated complications. Reported symptoms include nonspecific abdominal pain, dyspepsia, nausea, vomiting, gastrointestinal bleeding, and features of gastric outlet or small bowel obstruction, mirroring the abdominal discomfort observed in our first patient and the obstructive presentation in our second case [3,4]. Ectopic pancreas may also serve as a pathological lead point for intussusception, particularly in small bowel lesions, as demonstrated in our second case [4]. Less commonly, complications such as pancreatitis arising within heterotopic tissue, cyst formation, and malignant transformation have been described [4,5].

Preoperative diagnosis remains challenging because the clinical and radiological features of ectopic pancreas overlap significantly with other subepithelial and intramural gastrointestinal lesions [2,6]. The principal differential diagnoses include GIST, NET, leiomyoma, duodenal adenocarcinoma, lymphoma, metastatic lymph nodes, and duplication cysts [2,3]. As highlighted by Rezvani et al., although certain imaging characteristics, such as a lobulated contour, central umbilication, or enhancement similar to pancreatic parenchyma, may suggest ectopic pancreas, these features are neither sensitive nor specific [6,7]. Similarly, Trifan et al. emphasised the frequent misidentification of gastric heterotopic pancreas as stromal tumours or other submucosal masses due to nonspecific endoscopic and imaging findings. Advanced imaging modalities, including CT, MRI, and endoscopic ultrasound (EUS), may improve lesion characterisation but often fail to provide definitive preoperative diagnosis [8].

The role of functional imaging is also noteworthy. FDG-PET may demonstrate avidity in malignant GISTs or other metabolically active lesions [9]. However, benign ectopic pancreatic tissue typically does not exhibit significant FDG uptake [2,6]. In our cases, PET imaging was negative, which reduced but did not eliminate concern for malignancy. In the second case, DOTATATE PET was additionally performed to exclude a NET, consistent with established imaging strategies for NET evaluation [10].

Given this overlap with malignant and premalignant conditions, histopathological confirmation remains the gold standard for definitive diagnosis. Imaging findings alone are often insufficient to confidently exclude malignancy [6]. Consequently, surgical resection is frequently undertaken both to establish a definitive diagnosis and to relieve symptoms arising from a suspected pathological lesion, while definitively excluding malignant disease, as illustrated in our first case [2].

The two cases presented illustrate the heterogeneous nature of ectopic pancreas. In the first case, the lesion presented as a subepithelial mass with indeterminate imaging characteristics, mimicking a neoplastic process and posing a diagnostic challenge despite multimodal evaluation. In contrast, the second case demonstrated an intraluminal lesion acting as a lead point for intussusception, resulting in an acute clinical presentation. These differences highlight the variable detectability on imaging and endoscopy, as well as the spectrum from nonspecific symptoms to overt obstructive pathology.

The variability in presentation across different anatomical sites further reinforces the heterogeneous nature of ectopic pancreas. Christodoulidis et al., in their review of gastric heterotopic pancreas, described lesions typically presenting as submucosal antral masses that may cause epigastric pain, nausea, or vomiting, yet often remain incidental findings [11]. Broader adult narrative reviews have similarly emphasised the protean manifestations of ectopic pancreas throughout the gastrointestinal tract, ranging from asymptomatic subepithelial nodules to obstructive or inflammatory complications [12]. In the pediatric population, as described by Matran et al., ectopic pancreatic tissue is uncommon but may present with bleeding, obstruction, or intussusception, again underscoring its diverse clinical spectrum [13].

Although malignant transformation is rare, adenocarcinoma arising within heterotopic pancreatic tissue has been documented in the literature [5]. The reported incidence remains low; however, the possibility of malignant degeneration contributes to the clinical dilemma when managing indeterminate subepithelial lesions [4,5]. This potential, combined with nonspecific imaging characteristics, explains why definitive diagnosis frequently relies on histopathological examination.

The cases presented here demonstrate the diverse clinical and radiological manifestations of ectopic pancreas, from a subepithelial duodenal lesion mimicking GIST to an intraluminal small bowel mass acting as a lead point for intussusception. This variability supports its characterisation as a “chameleon” of abdominal pathology and highlights the importance of maintaining a broad differential diagnosis when evaluating gastrointestinal masses.

## Conclusions

Ectopic pancreas should be considered an important differential diagnosis for subepithelial lesions of the duodenum and small bowel, as it may closely mimic GISTs and other neoplastic processes on imaging. Radiological and endoscopic findings are frequently inconclusive, and definitive diagnosis often relies on histopathological evaluation.

Given its diverse clinical presentations and overlapping imaging characteristics, ectopic pancreas represents a true “chameleon” of abdominal pathology. Multidisciplinary assessment is essential to balance the risks of malignancy against the morbidity of intervention. Surgical resection may be appropriate in selected cases, particularly when malignancy cannot be confidently excluded or when symptoms persist. Increased awareness of this entity, particularly in cases presenting as subepithelial lesions or unexplained small bowel obstruction, may improve diagnostic consideration, reduce uncertainty, and guide more tailored management strategies.
